# Exploring the Value of Quantitative Muscle Ultrasound in Neuromuscular Junction Disorders: A Pilot Study

**DOI:** 10.1002/mus.70361

**Published:** 2026-07-30

**Authors:** Artor Pogosean, Maarika Liik, Nens van Alfen, Anna Rostedt Punga

**Affiliations:** ^1^ Department of Medical Sciences, Clinical Neurophysiology Uppsala University Uppsala Sweden; ^2^ Clinical Neurophysiology Uppsala University Hospital Uppsala Sweden; ^3^ Department of Neurology and Clinical Neurophysiology, Clinical Neuromuscular Imaging Group, Donders Institute for Brain, Cognition, and Behavior Radboud University Medical Center Nijmegen the Netherlands

**Keywords:** myasthenia gravis, neuromuscular transmission, quantitative muscle ultrasound, repetitive nerve stimulation, single‐fiber electromyography

## Abstract

**Introduction/Aims:**

Quantitative muscle ultrasound (QMUS) is a validated technique for assessing muscle pathology, yet its role in disorders primarily affecting the neuromuscular junction (NMJ) remains unclear. This pilot study aimed to explore whether QMUS is associated with structural muscle changes in patients with acquired or genetic NMJ disorders.

**Methods:**

We conducted a retrospective study of 18 patients with confirmed NMJ disorders who underwent electrodiagnostic testing (EDx), including repetitive nerve stimulation (RNS) and/or stimulated single‐fiber electromyography (SFEMG), combined with QMUS. Patients were grouped into congenital myasthenic syndromes (CMS) and acquired autoimmune myasthenia (myasthenia gravis [MG] or Lambert‐Eaton myasthenic syndrome [LEMS]). QMUS scores were classified as normal, borderline, or abnormal based on muscle echogenicity *z*‐scores derived from a validated algorithm.

**Results:**

Abnormal EDx was present in 13 of 18 (72%) patients. Abnormal decrement (> 10%) was observed in 7/18 patients, and abnormal jitter in the orbicularis oculi muscle was found in 6/8 patients. QMUS demonstrated normal echogenicity in 14/18 (78%) patients, while 4/18 (22%) showed abnormal or borderline echogenicity, including 2/5 (40%) with generalized MG. All patients with borderline or abnormal QMUS findings had concomitant abnormal RNS and/or SFEMG.

**Discussion:**

Structural muscle changes, reflected by altered echogenicity, may occur in a subset of patients with NMJ disorders, although QMUS shows limited sensitivity compared with EDx. Future studies using standardized protocols, dynamic assessments, and targeted evaluation of clinically relevant muscles, such as facial and bulbar, are needed to determine whether QMUS may serve as a complementary tool for disease characterization or monitoring.

**Abbreviations**
AChR: acetylcholine receptor
CMS: congenital myasthenic syndrome(s)
EDx: electrodiagnostic(s)
LEMS: Lambert‐Eaton myasthenic syndrome
MG: myasthenia gravis
MuSK: muscle‐specific tyrosine kinase
NMD: neuromuscular disorders
NMJ: neuromuscular junction
QMUS: quantitative muscle ultrasound
Radboudumc: Radboud University Medical Center
RNS: repetitive nerve stimulation
SFEMG: single‐fiber electromyography

## 1 Introduction

Neuromuscular ultrasound, and particularly quantitative muscle ultrasound (QMUS), is increasingly used as a non‐invasive complement to electrodiagnostic (EDx) testing in the evaluation of neuromuscular disorders (NMDs) [1, 2, 3, 4]. QMUS enables objective assessment of muscle structure and pathology through quantitative analysis of muscle echogenicity and has demonstrated good diagnostic accuracy in primary NMDs [5], although this varies across techniques and disease entities.

In contrast, the role of QMUS in disorders that primarily impair the neuromuscular junction (NMJ), such as myasthenia gravis (MG), Lambert‐Eaton myasthenic syndrome (LEMS), and congenital myasthenic syndromes (CMS), remains unclear. Findings from other clinical contexts suggest QMUS can reveal structural changes linked to impaired neuromuscular transmission, such as muscle architecture and echogenicity alterations after botulinum toxin treatment [6, 7]. The EDx tests repetitive nerve stimulation (RNS) and single‐fiber electromyography (SFEMG) are well‐established diagnostic tools for NMJ disorders, particularly MG, with high sensitivity and specificity [8, 9, 10]. However, QMUS has rarely been explored in this context. Limited studies suggest potential ultrasound abnormalities in MG, including diaphragm fatigability and hemidiaphragm atrophy [11, 12].

In parallel, muscle biopsy studies have demonstrated heterogeneous structural abnormalities, including myopathic and neurogenic changes, across both acetylcholine receptor (AChR)‐ and muscle‐specific tyrosine kinase (MuSK) antibody seropositive MG and selected CMS subtypes [13, 14, 15, 16].

Against this background, we conducted a retrospective pilot study to explore whether QMUS findings are associated with EDx‐detected abnormalities in patients with NMJ disorders. We hypothesized that a subset of patients, particularly those with CMS, in whom NMJ dysfunction may be more pronounced and chronic, would demonstrate increased muscle echogenicity on QMUS, and that such findings would co‐occur with abnormalities on RNS and/or SFEMG.

## 2 Methods

### 2.1 Subjects

Inclusion criteria included all patients evaluated at Radboud University Medical Center (Radboudumc) between 2013 and 2024 who underwent QMUS as part of the routine neurophysiological work‐up for suspected NMD, in addition to RNS and/or stimulated SFEMG, and who later received a confirmed diagnosis of MG, LEMS, or CMS.

### 2.2 General Setup and Protocol of Neurophysiological Examinations

All examinations were performed at the Department of Neurology and Clinical Neurophysiology, Radboudumc, Nijmegen, the Netherlands. As this was a retrospective study, no predefined protocol was used. All patients underwent RNS and/or SFEMG and QMUS, and only data from the visit that included QMUS were analyzed. Local Radboudumc reference values were applied. RNS and SFEMG recordings were performed using a Synergy EDX system (Natus Medical Inc., Middleton, Wisconsin, USA), and ultrasound was performed using an Esaote MyLab Twice system with fixed settings (Esaote, Genoa, Italy).

#### 2.2.1 Repetitive Nerve Stimulation (RNS)

Supramaximal stimulation was maintained. Baseline trains of 10 stimuli at 3 Hz were delivered at rest and after voluntary maximum contraction for 30 s, or 20 Hz stimulation for 10 s, to test for an increment. Neuromuscular transmission failure was defined as a reproducible decrement of ≥ 10% in compound muscle action potential amplitude between the first and fourth responses in one or more muscles; in muscles showing a decrement, post‐activation increment was considered abnormal if ≥ 100% (borderline ≥ 60%). Typically, the trapezius, abductor digiti minimi, and nasalis muscles were tested.

#### 2.2.2 Single‐Fiber Electromyography (SFEMG)

Stimulated SFEMG targeted the frontal branch of the facial nerve at 10 Hz using a disposable monopolar needle. (28 G × 25 mm; Natus Medical Inc., Middleton, USA). Jitter was recorded in the orbicularis oculi with a disposable single‐fiber needle electrode (35 × 0.45 mm; Citadella, Italy). Filter settings: 1–10 kHz. Abnormality: mean consecutive difference > 30 μs or > 10% of fiber pairs exceeding jitter threshold.

#### 2.2.3 Quantitative Muscle Ultrasound (QMUS)

**FIGURE 1:**
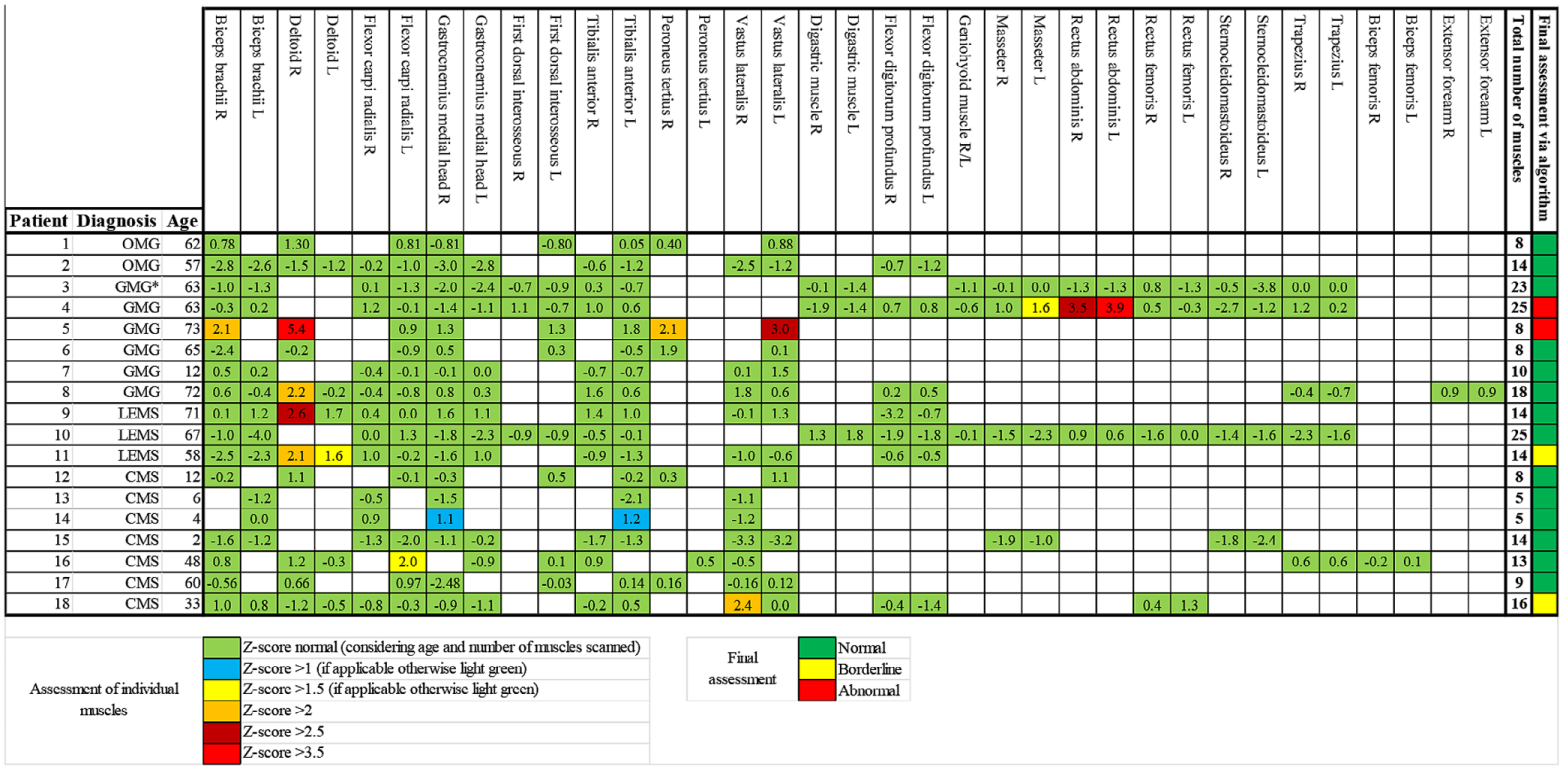
Schematic color map including all the echogenicity *z*‐score for each individual muscle in every patient. Color grading is based on assessment via the algorithm (Figure S1). Color table for assessment of individual muscles: Light green if *z*‐score normal (considering age and number of muscles scanned); *z*‐score > 1 blue if applicable (otherwise light green); *z*‐score > 1.5 yellow if applicable (otherwise light green); *z*‐score > 2 orange; *z*‐score > 2.5 maroon; *z*‐score > 3.5 red. Color table in final assessment: Dark green if normal, yellow if borderline and red if abnormal. Age at time of quantitative muscle ultrasound. CMS, congenital myasthenic syndrome; GMG*, oculobulbar myasthenia gravis; GMG, generalized myasthenia gravis (limb predominance); L, left; LEMS, Lambert‐Eaton myasthenic syndrome; OMG, ocular myasthenia gravis; R, right.

Standard QMUS protocols at Radboudumc include either limited screening protocols (typically 4 muscles) or extended protocols (typically ≥ 8 muscles). Muscle echogenicity was evaluated for all scanned muscles (5–25 per patient; Figure 1). Muscle diameter and echogenicity (0–255 arbitrary units, A.U.) were analyzed using QUMIA software (v.2.1, developed in‐house). The echogenicity was assessed using a validated algorithm [5, 17, 18] that depended on age (< 65 or ≥ 65 years), number of muscles examined (< 6 or > 6), and z‐score; the final assessment was concluded as “normal”, “borderline” (NMD neither confirmed nor refuted), or “abnormal” [5, 19] (Figure S1). Diameter was not included in the primary analysis due to low sensitivity and high inter‐individual variability. Local reference values adjusted for age, sex, and body mass index were used [20, 21, 22].

### 2.3 Statistical Analysis

Descriptive statistics were used due to the small, heterogeneous sample. Analyses were performed using Microsoft Excel 16.103 (Redmond, Washington, USA) and GraphPad Prism 10.6.1 (Boston, Massachusetts, USA).

## 3 Ethical Consideration

All patients consented to anonymized reuse of their data via their electronic health record. No additional ethics approval was required per local protocol.

## 4 Results

### 4.1 Subjects and General Findings

Eighteen patients were included. Final diagnoses were established according to disease‐specific guidelines, integrating clinical criteria with serology, genetics, and/or EDx. Eight patients were diagnosed with MG, three with LEMS, and seven with CMS. Most MG patients (6/8) and all LEMS patients had antibodies against AChRs or voltage‐gated calcium channels. CMS diagnoses were based on pathogenic genetic mutations, EDx, or characteristic clinical features, including treatment response (Table 1).

**TABLE 1:** Descriptive information of all patients included in this study.

Patient	Diagnosis	Further details	Sex	Age (y)			Disease duration (mo)	Height (cm)	Weight (kg)
				Diagnosis	QMUS	EDx			
1	OMG	AChR(+)	M	61	62	62	1	180	83
2	OMG	AChR(−)	M	50	57	50	92	178	91
3	GMG*	AChR(−)	M	63	63	63	4	172	67
4	GMG	AChR(+), lung cancer	M	64	63	63	48	176	64
5	GMG	AChR(+)	F	70	73	73	4	173	92
6	GMG	AChR(+)	M	66	65	65	17	189	102
7	GMG	AChR(+), thymoma	F	12.5	12	12	4	169	62
8	GMG	AChR(+), GVHD	M	70	72	72	19	172	67
9	LEMS	VGCC(+), small cell lung cancer	M	71	71	71	2	184	90
10	LEMS	VGCC(+)	M	66	67	67	12	175	85
11	LEMS	VGCC(+)	F	59	58	58	19	180	125
12	CMS	*CTNND2* deletion	M	12	12	12	44	142	39
13	CMS	No genetic cause in WES	F	6	6	6	72	119	20
14	CMS	No genetic cause in WES	M	5	4	4	58	110	20
15	CMS	No genetic cause in WES and OES	F	0.5	2	2	18	94	13
16	CMS	Homozygous *RAPSN* mutation	F	48	48	48	576	171	89
17	CMS	Heterozygous *CHRNA1* mutation	F	60	60	60	720	170	101
18	CMS	Heterozygous *RAPSN* mutation	M	29	33	33	348	179	80

*Note:* Height and weight at QMUS.

Abbreviations: AChR(+) acetylcholine receptor antibody seropositive; AChR(−), acetylcholine receptor antibody seronegative; *CHRNA1*, cholinergic receptor nicotinic alpha 1 subunit; CMS, congenital myasthenic syndrome; *CTNND2*, catenin delta 2; EDx, electrodiagnostics (specifically repetitive nerve stimulation and single‐fiber electromyography); F, female; GMG*, oculobulbar myasthenia gravis; GMG, generalized myasthenia gravis (limb involvement); GVHD, graft‐versus‐host disease after stem cell transplant for acute myeloid leukemia; LEMS, Lambert‐Eaton myasthenic syndrome; M, male; mo, months; OES, open exome screening; OMG, ocular myasthenia gravis; QMUS, quantitative muscle ultrasound; *RAPSN*, receptor associated protein of the synapse; VGCC(+), voltage‐gated calcium channel antibody seropositive; WES, whole exome sequencing; y, years.

All 18 patients underwent RNS, of whom seven showed abnormal decrement. SFEMG was performed in eight patients, with abnormal jitter observed in six. Thirteen patients (72%) showed abnormal neuromuscular transmission on RNS or SFEMG. QMUS findings were normal in all patients with ocular/oculo‐bulbar MG, while abnormal echogenicity was observed in 2 of 5 patients with generalized MG. Borderline QMUS findings were seen in 1 LEMS patient and in 1 CMS patient (Table 2; Figure 1).

**TABLE 2:** Summarized outcome from RNS, SF‐EMG and QMUS.

Patient	Diagnosis	RNS	SF‐EMG	EDx	QMUS	Muscles (*n*); Age (*y*)	Increased echogenicity (*z*‐score)^a^
1	OMG	Normal	Abnormal	Abnormal	Normal	8; 62	
2	OMG	Normal	Abnormal	Abnormal	Normal	14; 57	
3	GMG*	Normal	Abnormal	Abnormal	Normal	23; 63	
4	GMG	Normal	Abnormal	Abnormal	Abnormal	25; 63	Masseter L (1.6), RA L (3.9), RA R (3.5)
5	GMG	Normal	Abnormal	Abnormal	Abnormal	8; 73	BB R (2.1), Delt. R (5.4), PT R (2.1), VL L (3.0)
6	GMG	Normal	—	Normal	Normal	8; 65	
7	GMG	Abnormal	—	Abnormal	Normal	10; 12	
8	GMG	Normal	—	Normal	Normal	18; 72	
9	LEMS	Abnormal	—	Abnormal	Normal	14; 71	
10	LEMS	Abnormal	—	Abnormal	Normal	25; 67	
11	LEMS	Abnormal	—	Abnormal	Borderline	14; 58	Delt. L (1.6), Delt. R (2.1)
12	CMS	Normal	Abnormal	Abnormal	Normal	8; 12	
13	CMS	Abnormal	—	Abnormal	Normal	5; 6	
14	CMS	Normal	Normal	Normal	Normal	5; 4	
15	CMS	Normal	Normal	Normal	Normal	14; 2	
16	CMS	Normal	—	Normal	Normal	13; 48	
17	CMS	Abnormal	—	Abnormal	Normal	9; 60	
18	CMS	Abnormal	—	Abnormal	Borderline	16; 33	VL R (2.4)

Abbreviations: BB, biceps brachii muscle; CMS, congenital myasthenic syndrome; Delt., deltoid muscle; EDx, electrodiagnostics (summarized result for RNS and SF‐EMG in combination); GMG, generalized myasthenia gravis (limb predominance); GMG*, oculobulbar myasthenia gravis; L, left; LEMS, Lambert‐Eaton myasthenic syndrome; MG, myasthenia gravis; Muscles (n), number of muscles scanned at QMUS; OMG, ocular myasthenia gravis; PT, peroneus tertius muscle; QMUS, quantitative muscle ultrasound; R, right; RA, rectus abdominis muscle; RNS, repetitive nerve stimulation; SF‐EMG, single‐fiber electromyography; VL, vastus lateralis muscle; y, age in years at QMUS.

^a^ *z*‐score for all the muscles with increases echogenicity in all patients with abnormal or borderline QMUS result as evaluated with validated algorithm.

### 4.2 Muscle Ultrasound Details

Scanning extent and muscle selection varied across patients, with three undergoing extensive assessment (> 20 muscles). No consistent pattern of echogenicity or muscle diameter was observed across muscles or NMJ disease groups, with substantial inter‐ and intra‐individual variability in both muscle echogenicity and diameter (Figures S2 and S3). Overall, QMUS was abnormal in 2/18 (11%) and borderline in 2/18 (11%) patients.

### 4.3 Association Between Muscle Parameters and Neuromuscular Transmission

All patients with abnormal or borderline QMUS echogenicity also had abnormal RNS and/or SFEMG. No patient had abnormal QMUS in the absence of EDx abnormalities. Five patients (two with generalized MG, three with CMS) had normal results on all tests. Nine patients showed abnormal EDx findings and normal QMUS echogenicity.

Of the five patients with generalized MG, two had normal EDx and QMUS findings; two of the three with abnormal EDx results also had abnormal muscle echogenicity (Tables 1 and 2).

All ocular/oculo‐bulbar MG patients had abnormal SFEMG and normal QMUS; in two patients, facial or bulbar muscles were not scanned. One patient had QMUS 7 years after EDx.

All three LEMS patients had abnormal RNS, and one had borderline QMUS. In CMS (*N* = 7), one had borderline echogenicity, and six had normal QMUS. Three CMS patients also had normal RNS and/or SFEMG.

## 5 Discussion

In this study, abnormal or borderline muscle echogenicity on QMUS was present in a small subset of patients with neuromuscular transmission failure and EDx abnormalities. These findings suggest that secondary structural muscle changes may be present in some patients with NMJ disorders, despite these disorders being classified as functional synaptic conditions. If muscle architecture is compromised in a subset of patients with NMJ disorders, this may have implications for treatment and prognosis.

The observation that abnormal QMUS findings were always accompanied by abnormal RNS or SFEMG reinforces its potential as an adjunct rather than a standalone diagnostic tool.

Evidence from magnetic resonance imaging studies indicates that facial and bulbar muscles, particularly in MuSK antibody‐positive MG, can show atrophy and fatty replacement [23]. Facial and pharyngeal muscles involved in swallowing, along with the diaphragm, are clinically relevant in MG and other NMJ disorders and therefore should be prioritized in future ultrasound protocols. Incorporating dynamic assessments could further enhance diagnostic yield by capturing fatigue‐related changes or early structural alterations that static measurements miss [12, 24]. For example, M‐mode ultrasound of suprahyoid muscles has been successfully applied in dysphagia assessment [25], raising the possibility of similar approaches in NMJ disorders. Emerging ultrasound technologies may also expand diagnostic capabilities. Ultrafast ultrasound has recently enabled visualization of motor endplates in skeletal muscle [26]. If adapted for clinical use, such techniques could provide novel insights into NMJ pathology.

This study is limited by its retrospective design, a small and heterogeneous cohort, the absence of a control group, variable muscle selection, and potential clinical confounders, including comorbidities and incomplete treatment data. Nevertheless, these limitations reflect clinical practice and demonstrate the feasibility of integrating QMUS into routine neurophysiological assessments. Future prospective studies should employ standardized protocols, with targeted examination of clinically relevant muscles, and dynamic measurements to clarify its role and optimize its application in NMJ disorders.

## 6 Conclusion

This pilot study suggests that QMUS may be associated with secondary structural abnormalities in a subset of patients with NMJ disorders. While QMUS demonstrated limited sensitivity, it may provide complementary structural information in selected patients. Future studies using standardized, targeted, and dynamic ultrasound protocols focusing on involved muscles are needed to define the potential role of QMUS in the evaluation and monitoring of NMJ disorders.

## Author Contributions

**Artor Pogosean:** conceptualization, data curation, formal analysis, visualization, writing – original draft, writing – review and editing. **Maarika Liik:** conceptualization, writing – review and editing, supervision, resources. **Anna Rostedt Punga:** conceptualization, writing – original draft, supervision, project administration, writing – review and editing, funding acquisition, resources, validation. **Nens van Alfen:** conceptualization, data curation, formal analysis, methodology, investigation, supervision, writing – review and editing, validation.

## Funding

This work was supported by Region Uppsala.

## Ethics Statement

We confirm that we have read the Journal's position on issues involved in ethical publication and affirm that this report is consistent with those guidelines.

## Conflicts of Interest

The authors declare no conflicts of interest.

## Supporting information


**Figure S1:** Verified algorithm used to assess and score muscle ultrasound echogenicity.

**Figure S2:** Scatterplot of muscle diameter and echogenicity in the muscles examined in all patients (left and right sides plotted together). A. Patients with MG and LEMS. The first four scatterplots show muscle thickness (in cm) versus the corresponding z‐scores for each measurement in the muscles examined in all patients. The last plot on the row shows the distribution of echogenicity z‐score for the same four muscles. B. Patients with CMS. The first four scatterplots show muscle thickness (in cm) versus the corresponding z‐scores for each measurement in the muscles examined in all patients. The last plot on the row shows the distribution of echogenicity z‐score for the same four muscles. MG, myasthenia gravis; LEMS, Lambert‐Eaton myasthenic syndrome; CMS, congenital myasthenic syndrome.

**Figure S3:** Scatterplots of QMUS results. Parentheses show the number of individual muscles examined. **A**. Scatterplot of z‐score values of the diameter of all muscles examined in all patients (left and right side pooled together); the first plot displays results from all muscles, the second plot displays results from only the AQ‐group, and the third plot displays results from only the CMS‐group, respectively. **B**. Scatterplot of z‐score values of the echogenicity of all muscles examined in all patients (left and right side pooled together); the first plot displays results from all muscles, the second plot displays results from only the AQ‐group, and the third plot displays results from only the CMS‐group, respectively. AQ‐group, acquired myasthenia; CMS, congenital myasthenic syndrome.

## Data Availability

The data that support the findings of this study are available on request from the corresponding author. The data are not publicly available due to privacy or ethical restrictions.
